# Weekly external load distribution in football teams of different competitive levels

**DOI:** 10.5114/biolsport.2024.133668

**Published:** 2024-04-25

**Authors:** Diogo Coutinho, Diogo Oliveira1, Pedro Lisboa, Fábio Campos, Fábio Yuzo Nakamura1, Jorge Baptista1, Eduardo Abade

**Affiliations:** 1 Department of Sports Sciences and Physical Education, University of Maia, Maia, Portugal; 2 Research Center in Sports Sciences, Health Sciences and Human Development, CIDESD, 5000-801 Vila Real, Portugal; 3 Catapult Sports, Melbourne, Australia; 4 Performance Department, Futebol clube Famalicão SAD, Vila Nova de Famalicão, Braga, Portugal; 5 5Portugal Football School, Portuguese Football Federation, Oeiras, Portugal.; 6 Department of Sports Sciences, Exercise and Health, University of Trás-os-Montes and Alto Douro, Vila Real, Portugal

**Keywords:** Periodization, Distance-related variables, Acceleration and deceleration, Training load, Time-motion analyses

## Abstract

This study aimed to compare the microcycle load distribution between teams from different competitive levels. A total of 22 microcycles from one team of each competitive level (first division, 1^st^ DIV, n = 32 players; second division, 2^nd^ DIV, n = 23 players; third division, 3^rd^ DIV, n = 23 players) were monitored using GPS (10 Hz, Catapult). During the match, a higher number of high accelerations (i.e., > 3 m/s, per min) were found in the 3^rd^ DIV team compared to the 1^st^ and 2^nd^ DIV teams. On match day (MD) +1&+2, the 1^st^ DIV team covered more total (per min, *p* < 0.001) and high-speed running distance (HSR per min, *p* < 0.001 and *p* = 0.042, respectively) than both the 2^nd^ and 3^rd^ DIV teams. The 2^nd^ DIV team showed lower values in most distance-related variables (total distance covered per min, *p* < 0.001; running distance per min, *p* < 0.001; HSR per min, *p* < 0.001; and sprinting distance per min, *p* < 0.001) for both MD-4 and MD-3 compared to the 1^st^ and 3^rd^ DIV teams. In contrast, it showed higher sprinting distance per min (p < 0.001) on MD-2. In general, the 3^rd^ DIV team showed higher values in the number of high accelerations (per min, *p* < 0.001) across all sessions. These results suggest that distance-related variables may be a priority when planning microcycles for the 1^st^ DIV team, while accelerations are relevant for the 3^rd^ DIV team. A higher emphasis on external load during MD-2 by the 2^nd^ DIV team may explain the lower external loads across the microcycle.

## INTRODUCTION

Designing appropriate short- and mid-term plans for elite players poses a challenge for the technical staff, as it requires development of players’ collective movement abilities while adjusting training tasks to promote variations in the physical stimulus [1]. To address this, coaches have been using structured microcycles [2], which involve adopting similar weekly training session structures throughout the season, known as the match-day (MD) approach, but varying the target stimulus each day to ensure proper player development and recovery [3]. For instance, MD+1 or MD+2 is typically focused on recovering players who played more than 60 min in the previous match, while also simulating a similar period of activity for players with less playing time or no playing time [2]. During MD-4, coaches design agility, plyometric, and change-of-direction circuits, followed by rondos, sectorial tasks, and small-sided games (SSG) [3, 4] to target acceleration and deceleration patterns [5]. MD-3 emphasizes tasks that involve distance covered and distance covered at high speeds [3, 6], often employing large-sided games (LSG), collective sectorial tasks, and 11 v 11 conditioned games [2, 7]. As the upcoming game approaches, a tapering strategy is implemented, characterized by a decrease in external load [7, 8]. Consequently, MD-2 focuses on tasks that emphasize a number of sprints, combined with low-intensity activities to tactically prepare the team for the upcoming match [2, 4, 6]. Lastly, MD-1 typically has the lowest external load [9], incorporating rondos, reaction speed drills, and set pieces [2]. Despite this characterization of the microcycle structure, variations in external load may be expected when considering different training methodologies [3, 6], microcycles lengths [4, 10], contextual and cultural factors [11, 12], playing level and positional role [13].

Differences across playing levels have been extensively studied in youth players [14]. For instance, Coutinho et al. [15] compared MD+1, weekly sessions (excluding the first and last sessions of the week), and MD-1 among under-15, under-17, and under-19 teams. They found that the under-17 team covered more total distance per minute during all sessions than their counterparts. Moreover, the technical focus on the under-15 team seemed to result in lower variation between training days, while the higher emphasis on tactical preparation by the under-19 team limited players’ external load, particularly in the last session before the match. Similarly, a recent study compared an under-18 team with the corresponding senior team of the club and observed that the younger team performed more accelerations and decelerations in all training sessions [13]. Additionally, they covered more total distance during MD-4, MD-2, and MD-1, but displayed lower values of high-intensity and sprinting distance on MD-3 when compared to the senior team [13]. Although these studies have made valuable contributions, they primarily focus on the weekly load distribution in youth players. However, research exploring how senior teams from different playing levels manage their load distribution across the week is limited. From a scientific perspective, it is crucial to advance knowledge in this domain, particularly in relation to scouting and player selection from various competitive leagues. The inclusion of players from different leagues in higher-standard teams, and occasionally vice versa, raises important questions. Can a player from a lower-level league handle the external load expected in higher competitive levels? Additionally, it is essential to consider whether coaches from different competitive levels expose their players to training stimuli that can help them cope with the demands of matches. Understanding which external load may show more variability during the training week according to the competitive level will provide valuable insights into player development and performance. Consequently, the primary aim of this study is to characterize and identify differences in the weekly external load distribution among teams belonging to different playing levels. By shedding light on this aspect, this research aims to gain valuable insights to understand how teams of different competitive levels manage their training load distribution throughout the week.

## MATERIALS AND METHODS

### Experimental Approach to the Problem

This study intended to measure the differences in match and training sessions’ external load across different competitive leagues within the same country during the 2022-2023 season. A total of twentytwo training microcycles (∼7 microcycles per team, see Table 1) were collected from three teams (1^st^ DIV, n = 1; 2^nd^ DIV, n = 1 and 3^rd^ DIV, n = 1). The teams from the 1^st^ and 2^nd^ DIV participated in three national competitions (League Competition, Portugal Cup and League Cup), while the team from the 3^rd^ DIV participated in two national competitions (League Competition and Portugal Cup). While additional microcycles were collected (n = 2-3 per team), they were excluded as result of [5]: i) having less than 5 training days; ii) microcycles from congested fixtures; iii) having a day off between the match and MD-4; and iv) microcycles from weeks without any competitive fixture. In addition, individual training sessions focused on refining movement patterns, rehabilitation or recovery sessions were excluded from the analyses [8, 10]. All matches and sessions were monitored on natural grass pitches (i.e., length ∼100 m × ∼64 m), under similar weather conditions (i.e., all teams were located in a similar zone, maximum distance ∼75 km) and similar time periods (i.e., training sessions collected within a period from 9.00 a.m. to 12.00 a.m., while matches were played from 2.00 p.m. to 6.00 p.m.). While the duration of training tasks included the warm-up, main phase and cool down, data from matches included only the match information.

**TABLE 1:** Characteristics of training days.

	1st DIV	Duration (M ± SD)	Description
Match	8	97.31 ± 2.67	Match session that included all players’ who undertook at least 90-min of the match.

MD+1&+2	4	75.52 ± 8.39	Top up session loading players with less than 60-min of match play. Tasks included exercises performed in small to moderate sizes, such as ball possession related tasks, followed by speed finishing drills and ending with SSG.

MD-4	8	113.38 ± 24.28	Session characterized by small to medium spaces to promote high-intensity accelerations and decelerations while focusing on specific offensive and defensive principles of play (from 1 v 1 to 5 v 5).

MD-3	8	69.27 ± 9.06	Session dedicated to team collective play, based on large-sided games (e.g., 10 v 10, 10 v 8) to refine specific movement patterns (e.g., building up from the back). This mostly involved ball possession, sectorial and 11 v 11 conditioned tasks.

MD-2	8	65.41 ± 7.33	Tapering session, often composed by passing drills, specific movement patterns with low complexity (i.e., passive opposition) and finishing tasks.

MD-1	8	42.6 ± 6.99	Low-intensity session aiming to decrease load and foster a positive psychological state by using teambuilding activities, team strategical movement patterns and preparing match set-pieces.

**Training Day**	**2nd DIV**	**Duration (M ± SD)**	**Description**

Match	6	96.38 ± 4.07	Match session, that included all players’ that performed at least 90-min of the match.

MD+1&+2	5	65.37 ± 11.28	Top up session loading players with less than 60-min of match play. Accordingly, the players were introduced to BPG, followed by practicing shooting and finalization exercises and ending with SSG.

MD-4	6	76.77 ± 12.97	Session characterized using small to moderate spaces. Often the session started with BPG or SSG, progressing to intra-sectorial and inter-sectorial tasks to emphasize defensive performance.

MD-3	6	79.6 ± 5.97	Session in which the team tactical behaviour was the focus. Often the first task consisted in a LSG based on possession, progressing to defensive and offensive transitions using 8 v 8 and 10 v 10 LSG and ending with a competitive 11 v 11 game.

MD-2	6	68.63 ± 14.2	Session with major aim of tailoring the team offensive behaviour. Often tasks included passing patterns involving the 10 players, progressing to speed drills during finishing actions (e.g., 6 vs 3 + 1+ Gk) to develop crossing and finishing and ending with 11 v 11 game.

MD-1	6	62.14 ± 8.48	Session aiming to promote positive psychological state by using recreative and fun activities, often using reaction speed drills (e.g., reacting to colours with roles of catcher and runner), and ending with offensive and defensive set pieces.

**Training Day**	**3rd DIV**	**Duration (M ± SD)**	**Description**

Match	8	97.86 ± 3.77	Match session, that included all players’ that performed at least 90-min of the match.

MD+1&+2	5	73.99 ± 5.12	Top up session loading players with less than 60-min of match play. Training session consisted in exercises performed in small to moderate sizes. Session started with BPG, progressed to 1 v 1 and 2 v 2 situations and ended with SSG.

MD-4	6	76.04 ± 8.88	Training focused on acceleration and deceleration profiles by using BPG and SSG with 3 teams in small areas, and sectorial tasks consisting of 5 v 5 and 6 v 6 to foster defensive behaviour.

MD-3	8	92.36 ± 7.69	Training focused on reviewing the defensive patterns and developing the offensive organization in larger playing areas to stress distance covered. Often, it started with SSG and then progressed to tasks based on BPG in numerical superiority (9 v 9+2J) and both sectorial and collective tasks (from Gk+8 v 8+Gk to Gk+10 v 10+Gk).

MD-2	7	76.0 ± 8.49	Session that targets sprinting number and distance, while focusing on tapering to guarantee a proper recovery. Tasks often involved ball-passing patterns, BPG and SSG. Subsequently, the session was divided between attackers (i.e., wings and strikers) that practiced shooting and finalization, and defenders (i.e., centre-backs and fullbacks) who were planned to review the defensive principles.

MD-1	7	58.0 ± 7.95	Session started with light warm-up, followed by rondos, BPG and reaction speed tasks. The main session was focused on preparing both the offensive and defensive set pieces.

Note: MD+1&+2: 1 or 2 days after the match; MD-4: 4 days prior to next match; MD-3: 3 days prior to next match; MD-2: 2 days prior to the next match; MD-1: 1 day prior to the next match.

### Participants

A total of 78 professional outfield football players from Portugal Competitions during the 2022–2023 season participated in the present study (see Table 2, 1^st^ DIV, n = 32 players; 2^nd^ DIV, n = 23 players; 3^rd^ DIV, n = 23 players). While additional players engaged in the team training microcycles and competitive matches, only the players that took part in the full session or match during the data collection were considered for the planned analyses [6, 10]. In addition, the goalkeepers were excluded due to their restricted positioning on the pitch and different nature of training sessions [9, 16]. The study protocol followed the recommendations of the Declaration of Helsinki; however, following previous guidelines regarding data collection in elite sports [17], ethics committee clearance was not required.

**TABLE 2:** Players’ characterization according to their playing positions.

**Variables**	**1^st^ DIV**				
	Center Backs n = 8 Mean ± SD	Fullbacks n = 4 Mean ± SD	Midfielders n = 8 Mean ± SD	Wings n = 8 Mean ± SD	Strikers n = 4 Mean ± SD

Age (years)	21.29 ± 2.65	27.25 ± 4.19	24.49 ± 5.49	22.58 ± 2.94	27.65 ± 3.92
Height (m)	1.87 ± 0.02	1.79 ± 0.02	1.82 ± 0.04	1.82 ± 0.03	1.85 ± 0.04
Weight (kg)	79.71 ± 2.50	72.50 ± 5.20	75.5 ± 4.66	73.38 ± 4.00	75.25 ± 2.22
Playing Experience (years)	3.75 ± 3.06	8.00 ± 5.35	6.88 ± 5.33	5.00 ± 2.58	9.25 ± 5.32

**Variables**	**2^nd^ DIV**				
	Center Backs n = 4 Mean ± SD	Fullbacks n = 4 Mean ± SD	Midfielders n = 8 Mean ± SD	Wings n = 4 Mean ± SD	Strikers n = 3 Mean ± SD
Age (years)	27.83 ± 6.22	26.69 ± 3.58	25.72 ± 4.01	25.79 ± 2.85	29.56 ± 2.34
Height (m)	1.87 ± 2.50	1.79 ± 2.22	1.80 ± 8.63	1.72 ± 7.00	1.82 ± 3.79
Weight (kg)	83.00 ± 5.94	69.75 ± 2.87	70.63 ± 7.37	64.25 ± 8.42	78.67 ± 9.02
Playing Experience (years)	10.25 ± 5.50	8.45 ± 3.12	8.25 ± 3.30	9.00 ± 1.83	11.67 ± 1.53

**Variables**	**3^rd^ DIV**				
	Center Backs n = 4 Mean ± SD	Fullbacks n = 4 Mean ± SD	Midfielders n = 6 Mean ± SD	Wings n = 4 Mean ± SD	Strikers n = 5 Mean ± SD
Age (years)	26.10 ± 4.11	22.75 ± 1.00	23.29 ± 2.65	16.79 ± 11.16	26.52 ± 6.36
Height (m)	1.87 ± 0.01	1.78 ± 0.02	1.80 ± 0.08	1.77 ± 0.05	1.82 ± 0.03
Weight (kg)	82.10 ± 5.09	71.10 ± 2.33	74.32 ± 6.21	74.18 ± 9.81	76.12 ± 6.97
Playing Experience (years)	8.25 ± 4.11	5.75 ± 1.89	5.67 ± 2.80	4.50 ± 1.91	8.80 ± 5.81

Note: m = meters; kg = kilograms; n = number.

### Procedures

Player’s external load during each training session was monitored using portable 10 Hz Global Positioning System devices (GPS, Catapult, Vector S7, Catapult Sports, Melbourne, Australia). These units have been shown to be accurate in capturing players’ external load variables [18, 19]. To guarantee higher data reliability, and reduce inter-unit error, each player was assigned with a specific GPS unit that was used during all data collections [20, 21]. The GPS data from training sessions and matches were then downloaded to the manufacturer’s specific software (Catapult Openfield, version 3.10; Firmware 8.1).

The following variables were collected and expressed per minute [22–25]: total distance covered expressed in metres (m/min), distance covered while running (m/min, 14.4 km · h^−1^–19.7 km · h^−1^), high-speed running (HSR, m/min, > 19.8 km · h^−1^), sprinting distance (m/min, > 25.2 km · h^−1^), number of high accelerations (counts/min, > 3 m/s) and high decelerations (counts/min, > 3 m/s).

### Statistical Analysis

All data were preliminary tested for outliers, homogeneity and assumptions of normality and distribution through the Kolmogorov-Smirnov test. Descriptive statistics in tables and figures were expressed as mean (M) and standard deviation (SD). Further, a linear mixed model was used to compare players’ external load (i.e., total distance covered, running distance, HSR, sprinting distance, accelerations and decelerations) according to the competition level of the team (i.e., 1^st^ DIV, 2^nd^ DIV and 3^rd^ DIV), microcycle days (i.e., match, MD+1&+2, MD-4, MD-3, MD-2 and MD-1) and the interaction between competition level of the team and microcycle days. For that purpose, the competition level of the team and microcycle days were defined as categorical fixed effects, while individual players were considered as random effects. The pairwise comparisons between conditions were assessed using the Bonferroni *post-hoc* test. Complementarily, the magnitude of differences was quantified using the partial omega squared ($\omega_{\text{p}}^{2}$) and interpreted based on the following thresholds: small: < 0.01; medium: < 0.06; large, < 0.14. All statistical analyses were performed using the Jamovi Project software (Computer Software Version 2.3.21.0, 2023), with *p* < 0.05 as statistical significance.

## RESULTS

**FIG. 1:**
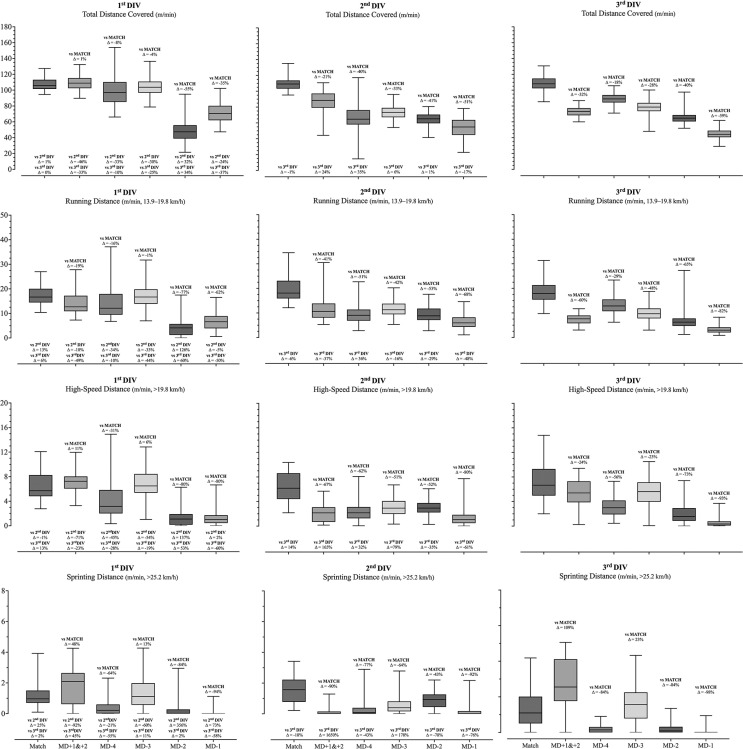
Descriptive (Mean, minimum and Maximum) data from the different moments (i.e., Match, MD+1&+2, MD-4, MD-3, MD-2 and MD-1) and the percentage difference between each moment and corresponding match and between different competitive levels for the distance-related variables.

**FIG. 2:**
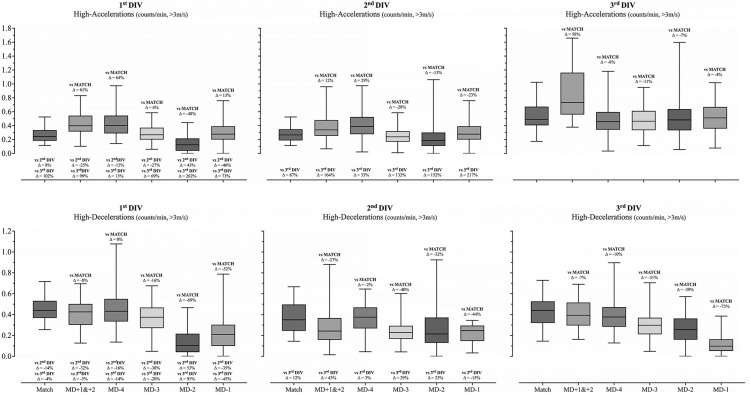
Descriptive (Mean, minimum and Maximum) data from the different moments (i.e., Match, MD+1&+2, MD-4, MD-3, MD-2 and MD-1) and the percentage difference between each moment and corresponding match and between different competitive levels for the acceleration and deceleration profile.

The descriptive and inferential data from the differences between teams’ playing level (i.e., 1^st^ DIV, 2^nd^ DIV and 3^rd^ DIV) at the different times (i.e., match, MD+1&+2, MD-4, MD-3, MD-2 and MD-1) are presented in Figure 1, Figure 2 and Table 3. The statistical analyses revealed statistically significant effects for the interaction (team × match day) in all variables. Interestingly, statistically significant effects in the match were only found for the number of accelerations (> 3 m/s), in which the 3^rd^ DIV team presented higher values than both 1^st^ and 2^nd^ DIV *(p* < 0.001). Despite this, statistically significant differences between competitive levels of teams were identified in all days of the microcycle. In addition, the 1^st^ DIV team reported higher distance-related variables on most days of the microcycle compared to their counterparts, with the exception of MD-2. Accordingly, the 1^st^ DIV team showed greater total distance covered (F = 122.7, *p* < 0.001, large effects) on M+1&+2, MD-4, MD-3 and MD-1 than both 2^nd^ DIV and 3^rd^ DIV teams. Similarly, greater running (F = 62.6, *p* < 0.001, moderate effects) and HSR distance (F = 62.4, *p* < 0.001, moderate effects) were found in the 1^st^ DIV team for MD+1&+2, MD-4 and MD-3 when compared to both 2^nd^ and 3^rd^ DIV teams. The 1^st^ DIV also accumulated higher running and HSR on MD-1 compared to the 3^rd^ DIV team. In contrast, these variables (total distance, running distance and HSR distance) were lower on MD-2 for the 1^st^ DIV team when compared to the 2^nd^ DIV team. Regarding the sprinting variable (F = 71.1, *p* < 0.001, moderate effects), the main statistically significant differences were found on MD-3 and MD-2, in which the 2^nd^ DIV team reported lower and higher values, respectively, than their counterparts. Another interesting result was the higher number of high accelerations (F = 29.5, *p* < 0.001, moderate effects) found in the 3^rd^ DIV team across all days of the microcycle when compared to the 1^st^ and 2^nd^ DIV teams. Accordingly, a higher number of acceleration events was noted in the 3^rd^ DIV team when compared to both 1^st^ and 2^nd^ DIV teams during MD+1& +2 and MD-1. Meanwhile, a higher number of accelerations on MD-4 and MD-2 were found in the 3^rd^ DIV team when compared to the 1^st^ DIV team, and on MD-3 when compared to the 2^nd^ DIV team.

**TABLE 3:** Descriptive statistics (M ± SD) from the external load according to the training days in relation to the team competitive level.

Variables		1^st^ DIV	2^nd^ DIV	3^rd^ DIV	*F (p-value)*	η2p (magnitude)	Post-hoc

		Mean ± SD	Mean ± SD	Mean ± SD			
**Total Distance Covered (m)**	Match	108.16 ± 8.34	109.73 ± 8.54	108.16 ± 8.76	122.7 (p < 0.001)	0.06 (large)
	MD+1|2	109.09 ± 8.68	86.54 ± 13.99	73.14 ± 6.94			1^st^ v 2^nd^; 1^st^ v 3^rd^; 2^nd^ v 3^rd^
	MD-4	99.60 ± 18.26	66.23 ± 15.36	89.15 ± 7.18			1^st^ v 2^nd^; 1^st^ v 3^rd^; 2^nd^ v 3^rd^
	MD-3	103.97 ± 9.95	73.19 ± 8.43	77.85 ± 9.71			1^st^ v 2^nd^; 1^st^ v 3^rd^
	MD-2	48.89 ± 14.03	64.65 ± 7.75	65.30 ± 7.32			1^st^ v 2^nd^; 1^st^ v 3^rd^
	MD-1	70.73 ± 12.69	53.73 ± 12.53	44.69 ± 5.80			1^st^ v 2^nd^; 1^st^ v 3^rd^; 2^nd^ v 3^rd^
**Running Distance (13.9–19.8 km/h, m)**	Match	17.61 ± 4.16	19.86 ± 5.39	18.68 ± 4.36	62.6 (p < 0.001)	0.03 (moderate)	
	MD+1|2	14.35 ± 4.78	11.70 ± 5.05	7.38 ± 1.97			1^st^ v 3^rd^; 2^nd^ v 3^rd^
	MD-4	14.78 ± 7.18	9.70 ± 4.01	13.24 ± 3.67			1^st^ v 2^nd^; 2^nd^ v 3^rd^
	MD-3	17.37 ± 4.89	11.59 ± 3.13	9.78 ± 3.22			1^st^ v 2^nd^; 1^st^ v 3^rd^
	MD-2	4.09 ± 3.11	9.24 ± 2.93	6.54 ± 2.90			1^st^ v 2^nd^; 1^st^ v 3^rd^; 2^nd^ v 3^rd^
	MD-1	6.68 ± 3.60	6.32 ± 2.52	3.31 ± 1.66			1^st^ v 3^rd^; 2^nd^ v 3^rd^
**High-Speed Running Distance (> 19.8 km/h, m)**	Match	6.41 ± 2.40	6.35 ± 2.42	7.21 ± 3.01	62.4 (p < 0.001)	0.03 (moderate)	
	MD+1|2	7.12 ± 1.91	2.08 ± 1.41	5.52 ± 2.16			1^st^ v 2^nd^; 1^st^ v 3^rd^; 2^nd^ v 3^rd^
	MD-4	4.41 ± 3.23	2.43 ± 1.63	3.20 ± 1.53			1^st^ v 2^nd^; 1^st^ v 3^rd^
	MD-3	6.80 ± 2.31	3.10 ± 1.43	5.53 ± 2.25			1^st^ v 2^nd^; 1^st^ v 3^rd^; 2^nd^ v 3^rd^
	MD-2	1.28 ± 1.28	3.03 ± 1.26	1.96 ± 1.52			1^st^ v 2^nd^
	MD-1	1.27 ± 1.19	1.30 ± 1.11	0.51 ± 0.58			
**Sprinting Distance (> 25.2 km/h, m)**	Math	1.25 ± 0.91	1.57 ± 0.96	1.28 ± 1.00	71.1 (p < 0.001)	0.04 (moderate)	
	MD+1|2	1.85 ± 1.18	0.15 ± 0.27	2.68 ± 1.58			1^st^ v 2^nd^; 1^st^ v 3^rd^; 2^nd^ v 3^rd^
	MD-4	0.46 ± 0.59	0.36 ± 0.64	0.21 ± 0.22			
	MD-3	1.42 ± 1.03	0.57 ± 0.55	1.58 ± 0.98			1^st^ v 2^nd^; 2^nd^ v 3^rd^
	MD-2	0.20 ± 0.39	0.89 ± 0.54	0.20 ± 0.28			1^st^ v 2^nd^; 2^nd^ v 3^rd^
	MD-1	0.07 ± 0.18	0.12 ± 0.24	0.03 ± 0.11			
**Accelerations (counts, > 3m/s)**	Math	0.27 ± 0.10	0.29 ± 0.10	0.54 ± 0.19	29.5 (p < 0.001)	0.02 (moderate)	1^st^ v 3^rd^; 2^nd^ v 3^rd^
	MD+1|2	0.43 ± 0.16	0.32 ± 0.19	0.85 ± 0.35			1^st^ v 3^rd^; 2^nd^ v 3^rd^
	MD-4	0.44 ± 0.18	0.37 ± 0.15	0.49 ± 0.22			2^nd^ v 3^rd^
	MD-3	0.28 ± 0.11	0.21 ± 0.10	0.48 ± 0.17			1^st^ v 3^rd^; 2^nd^ v 3^rd^
	MD-2	0.14 ± 0.11	0.20 ± 0.09	0.50 ± 0.24			1^st^ v 3^rd^; 2^nd^ v 3^rd^
	MD-1	0.30 ± 0.15	0.16 ± 0.08	0.52 ± 0.20			1^st^ v 2^nd^; 1^st^ v 3^rd^; 2^nd^ v 3^rd^
**Decelerations (counts, > 3m/s)**	Math	0.45 ± 0.11	0.38 ± 0.15	0.43 ± 0.13	28.5 (p < 0.001)	0.02 (moderate)	
	MD+1|2	0.41 ± 0.14	0.28 ± 0.16	0.40 ± 0.14			1^st^ v 2^nd^; 2^nd^ v 3^rd^
	MD-4	0.45 ± 0.17	0.37 ± 0.12	0.39 ± 0.15			
	MD-3	0.37 ± 0.13	0.23 ± 0.10	0.30 ± 0.11			1^st^ v 2^nd^
	MD-2	0.14 ± 0.11	0.21 ± 0.10	0.26 ± 0.12			1^st^ v 3^rd^
	MD-1	0.21 ± 0.14	0.14 ± 0.08	0.12 ± 0.09			1^st^ v 3^rd^

Note: 1^st^ v 2^nd^) Differences between 1^st^ DIV and 2^nd^ Div (p < 0.05); 1^st^ v 3^rd^) Differences between 1^st^ DIV and 3^rd^ Div (p < 0.05); 2^nd^ v 3^rd^) Differences between 2^nd^ DIV and 3^rd^ Div (p < 0.05).

## DISCUSSION

The aim of this study was to compare the load distribution within regular microcycles among teams from different competitive levels (1^st^ DIV, 2^nd^ DIV, and 3^rd^ DIV). The results revealed statistically significant differences between all teams on each day of the week, except for the match day, which only showed variations in the number of high accelerations.

Significant differences between teams for the match were only identified for accelerations. The literature in this domain has shown inconsistent results in the playing level comparisons. For instance, Mohr, Krustrup and Bangsbo [26] found that players competing at higher levels (i.e., big league and champions league) performed more high-speed running and sprinting than players from a moderate level (i.e., teams from the Danish first league). Similarly, higher-ranked teams from the 1^st^ DIV in the Spanish La Liga covered more total distance than those belonging to the 2^nd^ DIV [27]. In contrast, Bradley, Carling [28] found that players from the 2^nd^ DIV covered more highintensity running distance than those in the 1^st^ DIV. More recently, García-Calvo, Ponce-Bordón [29] found no differences when comparing 1^st^ and 2^nd^ DIV teams from Spain regarding high-metabolic load distance variables. The authors suggested that such results might be related to comparing team performance without considering players’ roles, and the same possibility could be considered in the present study. Considering that players’ roles are determinant for the accumulated match external load, further research may include comparing differences in match physical load of the different playing positions according to team competitive level. Nevertheless, 3^rd^ DIV teams presented a higher number of accelerations than both 1^st^ and 2^nd^ DIV teams. Teams from higher playing levels are more capable of maintaining ball possession, performing a higher number of frontal passes, and touching the ball more often [28], contributing to greater use of width [27]. In contrast, teams from lower competitive levels show higher values for headers, interceptions, and clearances [28]. These indicators may suggest that teams from lower competing levels may adopt a more “direct” playing style, emphasizing long balls and second balls, which can lead to more changes in ball possession between teams. As a result, players may need to accelerate often to press the opposition, move close to the ball location, or even explore possible counterattacks.

In this study, the analysis from MD+1&+2 sessions considered only players who performed the top-up session (i.e., players who were not exposed to a minimum of 60 min of the match) to better understand the session demands [2]. Consequently, higher values for distance-related variables were found for the 1^st^ DIV team, while the 3^rd^ DIV team showed higher values for acceleration. Non-starters (i.e., substituted players) seems to cover less than 40% running compared to starting players [30]. Accordingly, 1^st^ DIV teams seem to recognize this difference, and consequently, emphasize the distance covered for non-starters in the first session. However, a previous study exploring the differences in training load between starters and non-starters throughout an entire competitive season from an elite team found no differences in players’ training load distribution [31]. This evidence suggests that coaches from higher competitive levels may be more aware of the crucial role of compensatory strategies for non-starter players. The availability of resources also plays a role, as teams from higher competitive levels often count on “alternative” teams such as B or under-23 teams, which allows them to have more players to stress players’ physical load using game-based situations. On the other hand, teams from lower levels may be more limited in having additional players during training sessions, leading them to adopt the use of SSG, which often induce a higher number of accelerations compared to match formats involving more players [32]. While it is possible that coaches from lower competitive levels use SSG more often due to the lower number of available players, especially during MD+1&+2, it is also plausible that coaches from teams of such levels are aware of the matches’ physical demands. Therefore, they design specific tasks to prepare the players for the actual competitive demands. In fact, the results from the 3^rd^ DIV team showed a higher number of accelerations throughout the entire week when compared to both 1^st^ and 2^nd^ DIV teams. Since differences between competitive levels during the match in this study were only found for high accelerations, it seems to suggest that coaches from lower levels adjust the weekly training loads to match the competition demands. Despite that, it is important to note that players with more playing time across the season are likely to manifest higher accumulative weekly load compared to players who are less frequently selected [33]. Consequently, further research exploring different strategies on how to decrease the differences between line-up players, substitutes and reserves is required, mainly when considering MD+1&+2, MD-1 and MD [33].

The MD-4 session is often focused on developing intra-sectorial movement patterns within the team, such as movement coordination within the defensive sector while emphasizing neuromuscular actions (e.g., changes of direction) [2, 34]. Coaches from all competitive levels in this study seem to recognize the importance of neuromuscular overload on MD-4, as differences in acceleration were only evident between the 2^nd^ DIV and 3^rd^ DIV teams, with higher values for the latter. In fact, results from a diversity of backgrounds had shown a higher emphasis on acceleration and deceleration on MD-4, in which coaches uses SSG to emphasize such external load metrics [7].

In contrast to MD-4, MD-3 is often focused on the total distance covered and distance covered at high speed [6]. Coaches use larger formats of the match and include a higher number of players to develop inter-player and collective team movement patterns. However, a curious observation in the 2^nd^ DIV team was that it reported lower values for most variables during the first three training sessions (i.e., MD+1&+2, MD-4, and MD-3), contradicting the results found in most studies exploring microcycle external load distribution [4, 5, 11] and ‘periodization’ models in team sports [1, 4]. Nevertheless, coaches’ main priority during the competitive period is refining the team’s technical and tactical behaviour [8], and different pedagogical approaches may emerge as a result of the coaches’ profiles. For example, one coach may use short-period tasks to emphasize intensity and use rest periods to provide feedback, while another coach may adopt more continuous tasks and stop more often to provide tactical rearrangements of the team, which could impact their external load profile [15]. Interestingly, the 1^st^ DIV coach adopted the longer session on MD-4, which may support the above suggestion. Coaches from higher levels are expected to possess a deeper understanding of the match, enabling them to identify and correct team errors more precisely. Hence, they may adopt longer sessions to use the rest periods to adjust the team’s positioning. Additionally, the higher values for the distance-related variables on this day may support the suggestion that the coach from the 1^st^ DIV used the stoppages to provide feedback, contributing to differences mainly in the total distance covered.

The 2^nd^ DIV teams reported lower values of external load in most of the previous training sessions compared to both 1^st^ and 3^rd^ DIV teams. In contrast, higher values of distance covered at high intensities were identified on MD-2, and thus it is possible that the technical staff decided to induce a high stimulus to expose the players to an appropriate level of intensity, which has been found to be crucial in preventing injuries [35]. Coaches may adopt different methodological approaches based on the team’s characteristics. For example, when comparing teams from Portugal and the Netherlands belonging to the 2^nd^ DIV, Clemente, Owen [11] found that the Portuguese team covered more distance in the first two sessions (∼73% and ∼62% more), while covering less in the last (∼20 less). The same authors also found that the Portuguese team performed a higher number of sprints. Thus, it is possible that, like the acceleration and deceleration profile identified in the 3^rd^ DIV team, sprinting is a parameter that characterizes 2^nd^ DIV teams. For instance, coaches of 2^nd^ DIV teams may use transition-based tasks, crossing, and finishing actions on MD-2 [36], which may contribute to the higher sprinting demands.

A decrease in all external load parameters is evident on MD-1, in line with observations in research exploring weekly load distribution in youth academies [7] and semi-professional players [9]. However, despite this general decrease, statistically significant differences were still identified between competitive levels. For example, the 3^rd^ DIV team reported higher values for the number of high accelerations than both the 1^st^ and 2^nd^ DIV teams. While it might be expected for there to be a decrease in players’ acceleration profile as the microcycle progresses, a previous study found a constant distribution of acceleration volume across the microcycle [9]. This suggests that while the increase in accelerations throughout the week may help players be better prepared for the match demands, it could also lead to excessive fatigue [9]. It is worth noting that the 3^rd^ DIV team reported using SSG during all sessions of the week (see Table 1), which may have contributed to the high values in accelerations for all days of the microcycle [6, 32]. SSG are known to induce a higher number of accelerations due to the nature of the match format and the increased involvement of players in quick changes of direction and intense actions [32]. As a result, the frequent use of SSG by the 3^rd^ DIV team could explain the higher number of accelerations observed throughout the microcycle.

While this study provides important insights for coaches and sports practitioners, there are several limitations that should be acknowledged. Firstly, the analysis in this study is based on data from only one team from each competitive level, and thus the results from the external load may rely on coaches’ specific training methodology. In addition, players’ role within and between the team may also impact the present findings, and thus further research may consider including playing position as a covariate. These limitations may restrict the generalizability of the findings, as load distribution across the week can vary based on the training methodology employed by different teams [3, 6]. Including multiple teams from each competitive level would strengthen the study’s inferences and provide a more comprehensive understanding of load distribution patterns. Actually, programming characteristics of the technical staff might explain some of the differences reported herein, and they might not be solely related to the playing level. Additionally, previous research has highlighted that a team’s weekly external load may decrease when facing high-level opposition [12]. This factor was not considered in the present study, which could impact the load distribution within the microcycle. Future studies should account for the influence of the level of opposition on load distribution to gain a more comprehensive understanding of how teams adjust their training load based on the upcoming match difficulty.

## CONCLUSIONS

The results of this study revealed significant differences in microcycle load distribution between different competitive levels. Interestingly, only the number of high accelerations revealed statistically significant differences during the match, with higher values reported by the 3^rd^ DIV team. In contrast, there were substantial differences in the weekly load distribution, suggesting that different ‘periodization’ models emerge based on the competitive level. This may reflect varying coaching philosophies, training methodologies, playing styles, and contextual factors such as previous match scores and the quality of subsequent match opponents. Nonetheless, coaches, sports scientists, and strength and conditioning coaches can use these findings to obtain comparative values across different playing levels.

## References

[1] Los Arcos A, Mendez-Villanueva A, Martínez-Santos R. In-season training periodization of professional soccer players. Biol Sport. 2017; 34(2):149–55. Epub 20170101. doi: 10.5114/biolsport.2017.64588 PubMed PMID: 28566808; PubMed Central PMCID: . 28566808 PMC5424454

[2] Martin-Garcia A, Gomez Diaz A, Bradley PS, Morera F, Casamichana D. Quantification of a Professional Football Team’s External Load Using a Microcycle Structure. J Strength Cond Res. 2018; 32(12):3511–8. doi: 10.1519/JSC.0000000000002816. PubMed PMID: 30199452. 30199452

[3] Buchheit M, Lacome M, Cholley Y, Simpson BM. Neuromuscular Responses to Conditioned Soccer Sessions Assessed via GPS-Embedded Accelerometers: Insights Into Tactical Periodization. Int J Sports Physiol Perform. 2018; 13(5):577–83. Epub 20180522. doi: 10.1123/ijspp.2017-0045. PubMed PMID: 28872370. 28872370

[4] Oliva-Lozano JM, Gómez-Carmona CD, Fortes V, Pino-Ortega J. Effect of training day, match, and length of the microcycle on workload periodization in professional soccer players: a full-season study. Biol Sport. 2022; 39(2):397–406. Epub 20210601. doi: 10.5114/biolsport.2022.106148. PubMed PMID: 35309541; PubMed Central PMCID: . 35309541 PMC8919886

[5] Akenhead R, Harley JA, Tweddle SP. Examining the External Training Load of an English Premier League Football Team With Special Reference to Acceleration. J Strength Cond Res. 2016; 30(9):2424–32. Epub 2016/01/29. doi: 10.1519/jsc.0000000000001343. PubMed PMID: 26817740. 26817740

[6] Castillo D, Raya-González J, Weston M, Yanci J. Distribution of External Load During Acquisition Training Sessions and Match Play of a Professional Soccer Team. J Strength Cond Res. 2021; 35(12):3453–8. doi: 10.1519/jsc.0000000000003363. PubMed PMID: 31469765. 31469765

[7] Douchet T, Paizis C, Carling C, Cometti C, Babault N. Typical weekly physical periodization in French academy soccer teams: a survey. Biol Sport. 2023; 40(3):731–40. Epub 20221014. doi: 10.5114/biolsport.2023.119988. PubMed PMID: 37398965; PubMed Central PMCID: . 37398965 PMC10286623

[8] Malone JJ, Di Michele R, Morgans R, Burgess D, Morton JP, Drust B. Seasonal training-load quantification in elite English premier league soccer players. Int J Sports Physiol Perform. 2015; 10(4):489–97. Epub 20141113. doi: 10.1123/ijspp.2014-0352. PubMed PMID: 25393111. 25393111

[9] Swallow WE, Skidmore N, Page RM, Malone JJ. An examination of in-season external training load in semi-professional soccer players: considerations of one and two match weekly microcycles. Int J Sports Sci Coach. 2021; 16(1):192–9. doi: 10.1177/1747954120951762.

[10] Oliveira R, Brito J, Martins A, Mendes B, Calvete F, Carriço S, et al. In-season training load quantification of one-, twoand three-game week schedules in a top European professional soccer team. Physiol Behav. 2019; 201:146–56. Epub 20181106. doi: 10.1016/j.physbeh.2018.11.036. PubMed PMID: 30529511. 30529511

[11] Clemente FM, Owen A, Serra-Olivares J, Nikolaidis PT, van der Linden CMI, Mendes B. Characterization of the Weekly External Load Profile of Professional Soccer Teams from Portugal and the Netherlands. J Hum Kinet. 2019; 66:155–64. doi: 10.2478/hukin-2018-0054. PubMed PMID: 30988849. 30988849 PMC6458578

[12] Chena M, Morcillo JA, Rodríguez-Hernández ML, Zapardiel JC, Owen A, Lozano D. The Effect of Weekly Training Load across a Competitive Microcycle on Contextual Variables in Professional Soccer. Int J Environ Res Public Health. 2021; 18(10). Epub 20210511. doi: 10.3390/ijerph18105091. PubMed PMID: 34064978; PubMed Central PMCID: . 34064978 PMC8151593

[13] Morgans R, Rhodes D, Teixeira J, Modric T, Versic S, Oliveira R. Quantification of training load across two competitive seasons in elite senior and youth male soccer players from an English Premiership club. Biol Sport. 2023:1197–205. doi: 10.5114/biolsport.2023.126667. 37867738 PMC10588577

[14] Abade EA, Gonçalves BV, Leite NM, Sampaio JE. Time-motion and physiological profile of football training sessions performed by under-15, under-17 and under-19 elite Portuguese players. Int J Sports Physiol Perform. 2014; 9(3):463–70. doi: 10.1123/ijspp.2013-0120. PubMed PMID: 23920425. 23920425

[15] Coutinho D, Goncalves B, Figueira B, Abade E, Marcelino R, Sampaio J. Typical weekly workload of under 15, under 17, and under 19 elite Portuguese football players. J Sports Sci. 2015; 33(12):1229–37. doi: 10.1080/02640414.2015.1022575. PubMed PMID: WOS:000353402200004. 25789549

[16] White A, Hills SP, Cooke CB, Batten T, Kilduff LP, Cook CJ, et al. Match-Play and Performance Test Responses of Soccer Goalkeepers: A Review of Current Literature. Sports Med. 2018; 48(11):2497–516. doi: 10.1007/s40279-018-0977-2. PubMed PMID: 30144021. 30144021

[17] Winter EM, Maughan RJ. Requirements for ethics approvals. J Sports Sci. 2009; 27(10):985. doi: 10.1080/02640410903178344. 19847681

[18] Scott MT, Scott TJ, Kelly VG. The Validity and Reliability of Global Positioning Systems in Team Sport: A Brief Review. J Strength Cond Res. 2016; 30(5):1470–90. doi: 10.1519/jsc.0000000000001221. PubMed PMID: 26439776. 26439776

[19] Bastida Castillo A, Gómez Carmona CD, De la Cruz Sánchez E, Pino Ortega J. Accuracy, intraand inter-unit reliability, and comparison between GPS and UWB-based position-tracking systems used for time-motion analyses in soccer. Eur J Sport Sci. 2018; 18(4):450–7. Epub 20180131. doi: 10.1080/17461391.2018.1427796. PubMed PMID: 29385963. 29385963

[20] Castellano J, Casamichana D, Calleja-González J, Román JS, Ostojic SM. Reliability and Accuracy of 10 Hz GPS Devices for Short-Distance Exercise. J Sports Sci Med. 2011; 10(1):233–4. Epub 20110301. PubMed PMID: 24137056; PubMed Central PMCID: . 24137056 PMC3737891

[21] Jennings D, Cormack S, Coutts AJ, Boyd LJ, Aughey RJ. Variability of GPS units for measuring distance in team sport movements. Int J Sports Physiol Perform. 2010; 5(4):565–9. doi: 10.1123/ijspp.5.4.565. PubMed PMID: 21266740. 21266740

[22] Clemente FM, Rabbani A, Conte D, Castillo D, Afonso J, Truman Clark CC, et al. Training/Match External Load Ratios in Professional Soccer Players: A Full-Season Study. Int J Environ Res Public Health. 2019; 16(17):3057. PubMed PMID. doi: 10.3390/ijerph16173057. 31443592 PMC6747517

[23] Gregson W, Drust B, Atkinson G, Salvo VD. Match-to-Match Variability of High-Speed Activities in Premier League Soccer. Int J Sports Med. 2010; 31(04):237–42. Epub 20100215. doi: 10.1055/s-0030-1247546. 20157871

[24] Ade JD, Harley JA, Bradley PS. Physiological response, time-motion characteristics, and reproducibility of various speed-endurance drills in elite youth soccer players: small-sided games versus generic running. Int J Sports Physiol Perform. 2014; 9(3):471–9. doi: 10.1123/ijspp.2013-0390. 24509482

[25] Clemente FM, Silva R, Castillo D, Los Arcos A, Mendes B, Afonso J. Weekly Load Variations of Distance-Based Variables in Professional Soccer Players: A Full-Season Study. Int J Environ Res Public Health. 2020; 17(9):3300. PubMed PMID. doi: 10.3390/ijerph17093300. 32397398 PMC7246436

[26] Mohr M, Krustrup P, Bangsbo J. Match performance of high-standard soccer players with special reference to development of fatigue. J Sports Sci. 2003; 21(7):519–28. Epub 2003/07/10. doi: 10.1080/0264041031000071182. PubMed PMID: 12848386. 12848386

[27] Castellano J, Casamichana D. What are the differences between first and second divisions of Spanish football teams?. Int J Perform Anal Sport. 2015; 15(1):135–46. doi: 10.1080/24748668.2015.11868782.

[28] Bradley PS, Carling C, Gomez Diaz A, Hood P, Barnes C, Ade J, et al. Match performance and physical capacity of players in the top three competitive standards of English professional soccer. Hum Mov Sci. 2013; 32(4):808–21. doi: 10.1016/j.humov.2013.06.002. 23978417

[29] García-Calvo T, Ponce-Bordón JC, Pons E, López Del Campo R, Resta R, Raya-González J. High metabolic load distance in professional soccer according to competitive level and playing positions. PeerJ. 2022; 10:e13318. Epub 20220920. doi: 10.7717/peerj.13318. PubMed PMID: 36157060; PubMed Central PMCID: . 36157060 PMC9504445

[30] Young WB, Newton RU, Doyle TL, Chapman D, Cormack S, Stewart G, Dawson B. Physiological and anthropometric characteristics of starters and non-starters and playing positions in elite Australian Rules Football: a case study. J Sci Med Sport. 2005; 8(3):333–45. doi: 10.1016/s1440-2440(05)80044-1. PubMed PMID: 16248474. 16248474

[31] Oliveira R, Palucci Vieira LH, Martins A, Brito JP, Nalha M, Mendes B, Clemente FM. In-Season Internal and External Workload Variations between Starters and Non-Starters-A Case Study of a Top Elite European Soccer Team. Medicina (Kaunas). 2021; 57(7). Epub 20210623. doi: 10.3390/medicina57070645. PubMed PMID: 34201642; PubMed Central PMCID: . 34201642 PMC8306595

[32] Rebelo AN, Silva P, Rago V, Barreira D, Krustrup P. Differences in strength and speed demands between 4 v 4 and 8 v 8 small-sided football games. J Sports Sci. 2016; 34(24):2246–54. Epub 2016/06/10. doi: 10.1080/02640414.2016.1194527. PubMed PMID: 27278256. 27278256

[33] Casamichana D, Martín-García A, Díaz AG, Bradley PS, Castellano J. Accumulative weekly load in a professional football team: with special reference to match playing time and game position. Biol Sport. 2022; 39(1):115–24. Epub 20210304. doi: 10.5114/biolsport.2021.102924. PubMed PMID: 35173370; PubMed Central PMCID: . 35173370 PMC8805368

[34] Rey E, Corredoira FJ, Costa PB, Pérez-Ferreirós A, Fernández-Villarino MA. Acute effects of training load on contractile properties during a competitive microcycle in elite soccer players. Biol Sport. 2020; 37(2):157–63. Epub 20200330. doi: 10.5114/biolsport.2020.93041. PubMed PMID: 32508383; PubMed Central PMCID: . 32508383 PMC7249794

[35] Clemente FM, Silva R, Castillo D, Los Arcos A, Mendes B, Afonso J. Weekly Load Variations of Distance-Based Variables in Professional Soccer Players: A Full-Season Study. Int J Environ Res Public Health. 2020; 17(9). Epub 20200509. doi: 10.3390/ijerph17093300. PubMed PMID: 32397398; PubMed Central PMCID: . 32397398 PMC7246436

[36] Lewis G, Towlson C, Roversi P, Domogalla C, Herrington L, Barrett S. Quantifying volume and high-speed technical actions of professional soccer players using foot-mounted inertial measurement units. Plos One. 2022; 17(2):e0263518. doi: 10.1371/journal.pone.0263518. 35113962 PMC8812977

